# Insecticide-Mediated Up-Regulation of Cytochrome P450 Genes in the Red Flour Beetle (*Tribolium castaneum*)

**DOI:** 10.3390/ijms16012078

**Published:** 2015-01-19

**Authors:** Xiao Liang, Da Xiao, Yanping He, Jianxiu Yao, Guonian Zhu, Kun Yan Zhu

**Affiliations:** 1 Institute of Pesticide and Environmental Toxicology, Zhejiang University, Hangzhou 310029, China; E-Mails: liangxiaozju@126.com (X.L.); Zhugn@zju.edu.cn (G.Z.); 2 Department of Entomology, 123 Waters Hall, Kansas State University, Manhattan, KS 66506, USA; E-Mails: xd@cau.edu.cn (D.X.); yanping.he@gmail.com (Y.H.); jianxiu.yao@ag.tamu.edu (J.Y.)

**Keywords:** cytochrome P450, insecticides, *Tribolium castaneum*, up-regulation

## Abstract

Some cytochrome P450 (CYP) genes are known for their rapid up-regulation in response to insecticide exposures in insects. To date, however, limited information is available with respect to the relationships among the insecticide type, insecticide concentration, exposure duration and the up-regulated CYP genes. In this study, we examined the transcriptional response of eight selected CYP genes, including *CYP4G7*, *CYP4Q4*, *CYP4BR3*, *CYP12H1*, *CYP6BK11*, *CYP9D4*, *CYP9Z5* and *CYP345A1*, to each of four insecticides in the red flour beetle, *Tribolium castaneum.* Reverse transcription quantitative PCR (RT-qPCR) revealed that *CYP4G7* and *CYP345A1* can be significantly up-regulated by cypermethrin (1.97- and 2.06-fold, respectively), permethrin (2.00- and 2.03-fold) and lambda-cyhalothrin (1.73- and 1.81-fold), whereas *CYP4BR3* and *CYP345A1* can be significantly up-regulated by imidacloprid (1.99- and 1.83-fold) when 20-day larvae were exposed to each of these insecticides at the concentration of LC_20_ for 24 h. Our studies also showed that similar levels of up-regulation can be achieved for *CYP4G7*, *CYP4BR3* and *CYP345A1* by cypermethrin, permethrin, lambda-cyhalothrin or imidacloprid with approximately one fourth of LC_20_ in 6 h. Our study demonstrated that up-regulation of these CYP genes was rapid and only required low concentrations of insecticides, and the up-regulation not only depended on the CYP genes but also the type of insecticides. Our results along with those from previous studies also indicated that there were no specific patterns for predicting the up-regulation of specific CYP gene families based on the insecticide classification.

## 1. Introduction

Cytochrome P450 (CYP) genes constitute one of the largest gene superfamilies, with representatives in all living organisms, including bacteria, fungi, plants, and animals [1]. In insects, CYP enzymes are commonly involved in the metabolism of either endogenous or exogenous compounds. Although physiological significance of up-regulation or overexpression in insects is uncertain, the up-regulation is thought to provide versatility in environmental adaptation [2] or as a protective mechanism whereby the organism can detoxify xenobiotics [3]. Indeed, CYP-mediated detoxification is an important resistance mechanism that can cause a significantly high level of resistance to many insecticides in insect populations [4]. Besides detoxification, CYP genes are also involved in insect growth, development and nutrition [5]. It is believed that the diverse functions of cytochrome P450s are primarily due to the diversity of CYP genes [6]. To date, thousands of CYP genes in total have been identified in insects [7], and the number is still growing rapidly as more insect genomes are sequenced [8].

The up-regulation of CYP genes mediated by insecticides and other xenobiotic compounds have been reported in many insect species (references as presented in Table 1). The availability of genome sequences in many insect species has facilitated the identification of new CYP genes and the characterization of up-regulated CYP genes at the genomic scale [9]. For example, several microarray-based studies on *Drosophila melanogaster* have identified xenobiotic-inducible CYP genes [10,11]. These genes belong to CYP3, CYP4 and mitochondrial clans. The use of microarrays on insecticide-resistant mosquitoes, including *Anopheles gambiae* [12], *Aedes aegypti* [13] and *Culex quinquefasciatus* [14], have collectively identified a relatively small number of up-regulated CYP genes after exposures of the mosquitoes to different concentrations of insecticides. More recently, Zhu *et al.* [15] identified six CYP genes up-regulated in deltamethrin-resistant strain (QTC279) of *Tribolium castaneum*. Among them, *CYP6BQ9*, a brain-specific gene, showed over 200-fold constitutive overexpression, and can be up-regulated when the insects were exposured to deltamethrin [16].

The up-regulation of CYP genes could potentially have a significant impact on insect’s ability to metabolize xenobiotics, which may lead to the detoxification of insecticides and even the development of insecticide resistance in the insect populations. To date, however, limited information is available with respect to the relationship between CYP genes and the type of insecticides. In addition, little is known about the level of the up-regulation of CYP genes in relation to the insecticide concentration and the exposure duration in insects. The objectives of this study were to: (1) evaluate transcriptional responses of eight representative CYP genes, including *CYP4G7*, *CYP4Q4*, *CYP4BR3*, *CYP12H1*, *CYP6BK11*, *CYP9D4*, *CYP9Z5* and *CYP345A1*, to four selected insecticides, including cypermethrin, permethrin, lambda-cyhalothrin and imidacloprid, in *T. castaneum*; (2) examine the up-regulation responses of different CYP genes in relation to their classification to see whether the up-regulation is restricted to certain specific CYP families, or whether the up-regulation can be mediated by different insecticides within the same class; and (3) investigate the effect of insecticide concentrations and exposure duration on the level of the CYP up-regulation. This study is expected to help researchers better evaluate and understand the transcriptional responses of CYP genes to different insecticides in insects, and also provide useful information for future research to evaluate the role of CYP genes in insecticide detoxification and resistance in insects.

**Table 1:** Comparisons of selected cytochrome P450 (CYP) genes from *T. castaneum* with those known to be up-regulated by chemicals and/or overexpressed in insecticide resistant strains of other insects.

*T. castaneum* CYP Genes	Most Similar CYP Genes Found in Other Insect Species by BLASTP Search				
	Species	CYP Genes	Identity (%) ^a^	Overexpression Related to Insecticide Resistance ^b^	Up-Regulation Mediated by Insecticides and Other Chemicals
*CYP12H1*	*M. domestica*	*CYP12A1*	37	Pyrethroids [11]	Pyrethroids [17]
	*D. melanogaster*	*CYP12D1*	37	DDT [18]	pyrethrum [19]
	*D. melanogaster*	*CYP12A4*	39	Lufenuron [20]	-
	*A. gambiae*	*CYP12F1*	36	DDT [12]	-
	*A. aegypti*	*CYP12F8*	40	-	Fluoranthene [21,22]
*CYP4G7*	*M. domestica*	*CYP4G2*	48	-	Permethrin [23]
	*B. germanica*	*CYP4G19*	51	Pyrethroids [24]	-
	*C. tentans*	*CYP4G33*	54	-	Atrazine [25]
	*B. mori*	*CYP4G25*	52	-	Diazinon, permethrin [26]
	*A. aegypti*	*CYP4G36*	51	-	Imidacloprid [27]
*CYP4BR3*	*A. gambiae*	*CYP4H15*	39	DDT [12]	-
	*A. aegypti*	*CYP4H28*	38	-	Permethrin [28]
	*C. pallens*	*CYP4H21*	37	Deltamethrin [29]	-
	*C. quinquefasciatus*	*CYP4H34*	38	Permethrin [14]	-
	*D. melanogaster*	*CYP4E2*	41	-	Phenobarbital, caffeine [30]
	*D. melanogaster*	*CYP4E3*	41	-	Phenobarbital, caffeine [30]
*CYP4Q4*	*M. sexta*	*CYP4M1*	44	Alkaloids, nicotine [31]	-
	*B. mori*	*CYP4M5*	43	-	Dichlorvos, deltamethrin [32]
	*H. armigera*	*CYP4M6*	43	Deltamethrin [33]	-
	*D. virgifera virgifera*	*CYP4AJ1*	45	Parathion, carbaryl [34]	-
*CYP6BK11*	*M. domestica*	*CYP6A36*	49	Pyrethroids [35]	-
	*D. melanogaster*	*CYP6A8*	46	DDT, malathion [36]	Phenobarbital [30,37]
	*A. gambiae*	*CYP6P3*	43	Permethrin [38]	-
	*A. gambiae*	*CYP6M2*	43	Permethrin [39]	-
	*A. aegypti*	*CYP6M11*	41	Deltamethrin [40]	Permethrin [21]
	*P. xylostella*	*CYP6BG1*	39	Cypermethrin [41]	Permethrin [5]
*CYP345A1*	*D. melanogaster*	*CYP6G1*	43	DDT, imidacloprid [42]	DDT, caffeine [37]
	*M. domestica*	*CYP6D3*	38	Pyrethroids [43]	Phenobarbital [44]
	*C. quinquefasciatus*	*CYP6F1*	37	Permethrin [45]	-
	*A. aegypti*	*CYP6AL1*	37	Fluoranthene [21]	-
	*H. zea*	*CYP6B8*	36	Cypermethrin [46]	Chlorogenic acid [47]
*CYP9D4*	*C. quinquefasciatus*	*CY9M10*	37	Permethrin [48]	-
	*H. armigera*	*CYP9A12*	46	Pyrethroids [49]	-
	*H. armigera*	*CYP9A14*	43	Pyrethroids [50]	-
	*A. aegypti*	*CYP9J27*	41	Pyrethroids [13]	-
	*A. aegypti*	*CYP9J32*	44	Pyrethroids [13]	-
*CYP9Z5*	*A. mellifera*	*CYP9Q1*	38	Acaricides [51]	-
	*B. mori*	*CYP9A19*	45	-	-
	*B. mori*	*CYP9A20*	45	-	Dichlorvos, deltamethrin [32]
	*D. melanogaster*	*CYP9F2*	41	Pyrethrum [19]	-

^a^ The identity level is based on the deduced amino acid sequence of each CYP gene in *T. castaneum* against that of other insect species; ^b^ The number(s) in the brackets refer to reference numbers listed at the end of the paper.

## 2. Results and Discussion

### 2.1. Phylogenetic Analysis of Deduced Amino Acid Sequences of T. castaneum CYP Genes

**Figure 1:**
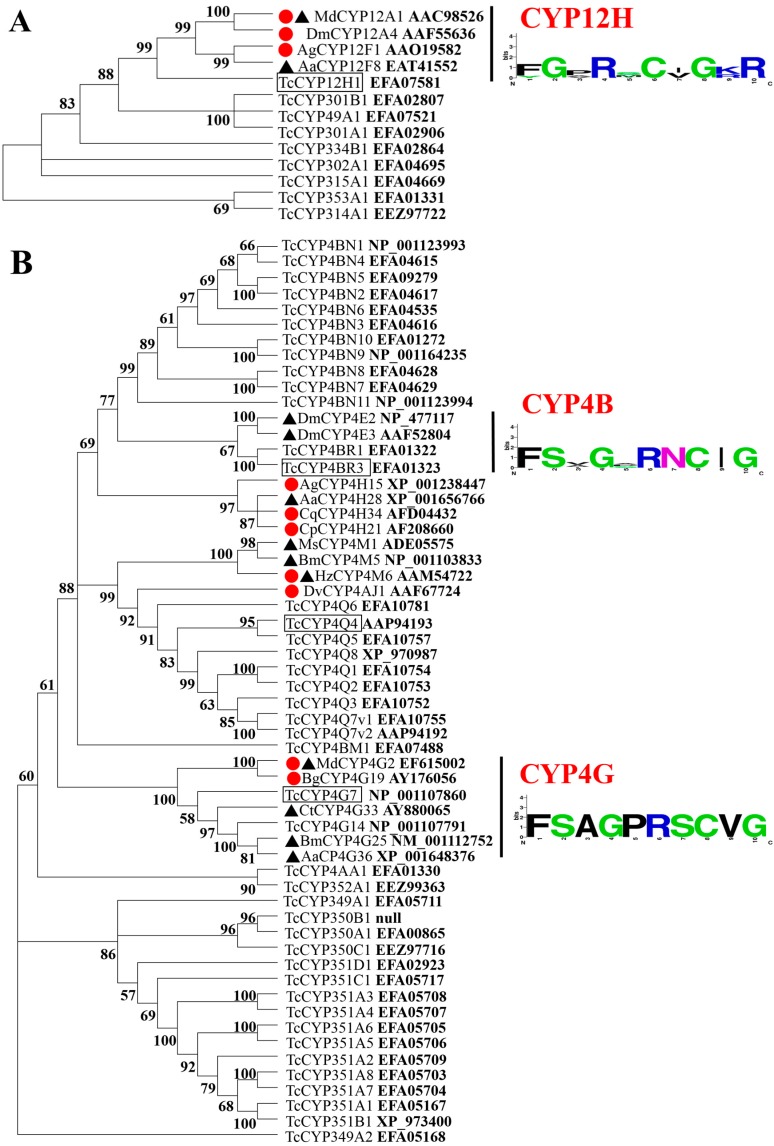

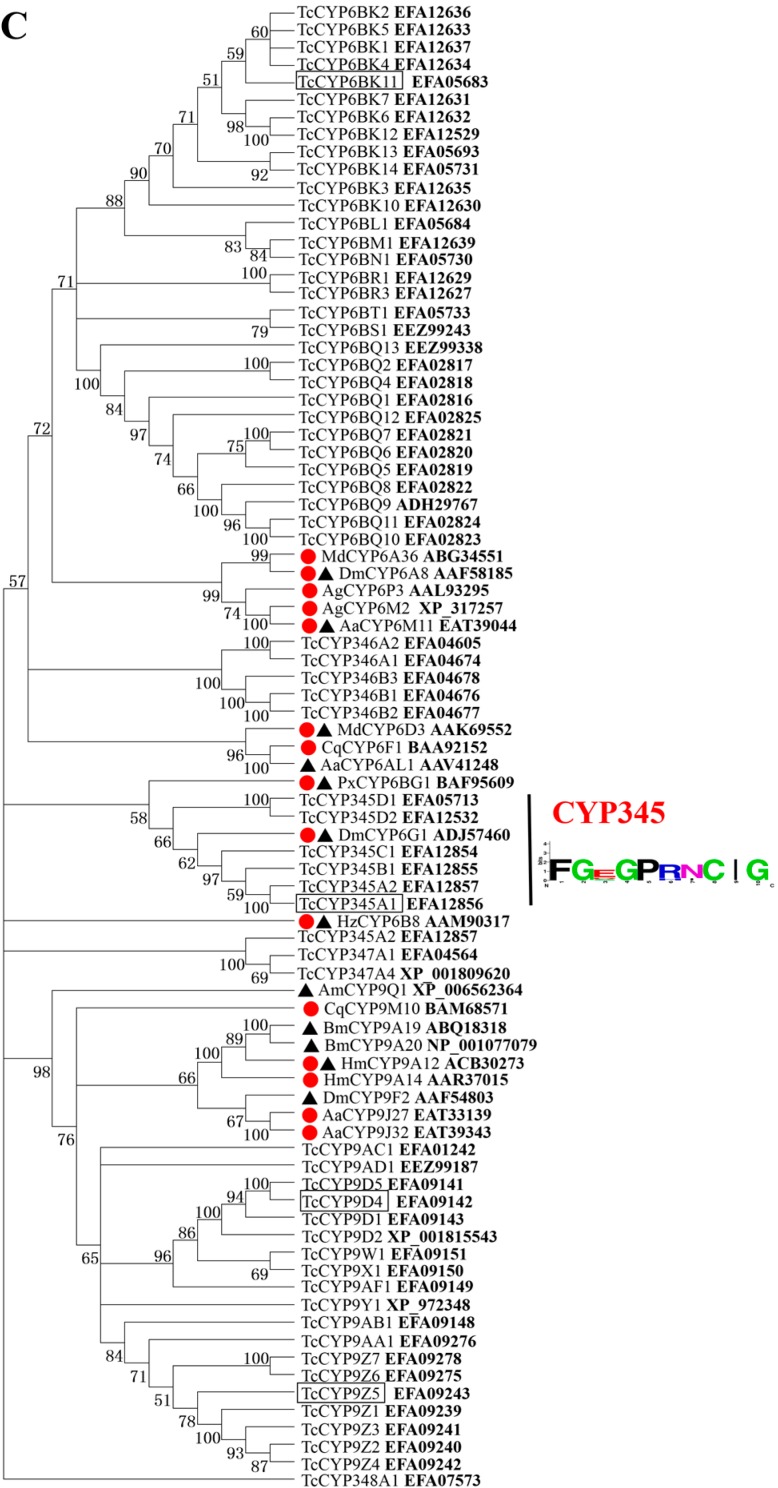
Neighbor-joining phylogenetic trees of three CYP clans. (**A**) mitochondrial; (**B**) CYP4; and (**C**) CYP3. The trees were constructed by using MEGA 5 based on the full-length amino acid sequences deduced from the cDNA or genomic DNA sequences of *T. castaneum* (Tc), *D. melanogaster* (Dm), *A. gambiae* (Ag), *Musca domestica* (Md), *A. aegypti* (Aa), *C. pipiens pallens* (Cp), *C. quinquefasciatus* (Cq), *Blattella germanica* (Bg), *Helicoverpa armigera* (Ha), *Diabrotica virgifera virgifera* (Dv), *Manduca sexta* (Ms), *Bombyx mori* (Bm), *H. Zea* (Hz), *Plutella xylostella* (Px), *Chironomus tentans* (Ct), and *Apis mellifera* (Am). The accession number of each gene from NCBI is shown in bold at the end of the gene name. All nodes have significant bootstrap support based on 3000 replicates. The trees were constructed with cut-off value of 50%. The CYPs known to be implicated in insecticide resistance and up-regulation were indicated with a black triangle and a red dot, respectively, or both. In addition, sequence logos, which were predicted by WebLogo tool (http://weblogo.berkeley.edu/logo.cgi), depicted the conservation of amino acid residues in CYP heme-binding motif of each clustered clade. The letter size is proportional to the degree of amino acid conservation. Eight CYPs selected for this study were boxed.

Phylogenetic analysis showed that CYP12H1 (Figure 1A), CYP4BR3 and CYP4G7 (Figure 1B) and CYP345 (Figure 1C) from *T. castaneum* were clustered in distinct clades with the CYPs from other insect species in the phylogenetic trees, and the heme-binding motifs of these clades were conserved. Noticeably, CYPs from CYP6 and CYP9 family were clustered in one clade within *T. castaneum* rather than with any other species. These clustered CYP genes may have similar roles, and therefore can help us select the representative CYP genes for further analyses in *T. castaneum*. For example, several genes in CYP6 and CYP9 gene families, which account for nearly half of all CYP genes in *T. castaneum*, have been implicated in the insecticide-mediated up-regulation and insecticide resistance [16].

### 2.2. Selection of CYP Genes for Studying Insecticide-Mediated Up-Regulation

We selected eight CYP genes from *T. castaneum* based on their representations in the phylogenetic trees and their similarities of amino acid sequences to those of other insect CYP genes known to be capable of up-regulation by insecticides (Table 1). The amino acid sequence identities of CYPs among *T. castaneum* and other insects range from 35%–54%, and seldom beyond 50% only if the comparisons were made within the same subfamily. For example, *CYP4G7* in *T. castaneum* shows the identities of 51% to *CYP4G19* from *B. germanica*, 54% to *CYP4G33* from *C. tentans*, 52% to *CYP4G25* from *B. mori*, and 51% to *CYP4G36* from *A. aegypti.* In order to select a manageable number of the CYP genes for subsequent analyses, the CYPs with the highest identities from eight major subfamilies (Table 1) were selected as representative genes. These genes include *CYP4G7*, *CYP4Q4*, *CYP4BR3*, *CYP12H1*, *CYP6BK11*, *CYP9D4*, *CYP9Z5* and *CYP345A1*. In the process of selecting the representative genes, we also considered the factor of which their homologous genes in other insect species have been reported in insecticide and other chemical-mediated up-regulation and/or insecticide resistance. This strategy has been successfully used by Poupardin *et al.* on *A. aegypti* [21].

### 2.3. Stage and Tissue Dependent Expression Patterns of Eight CYP Genes

**Figure 2:**
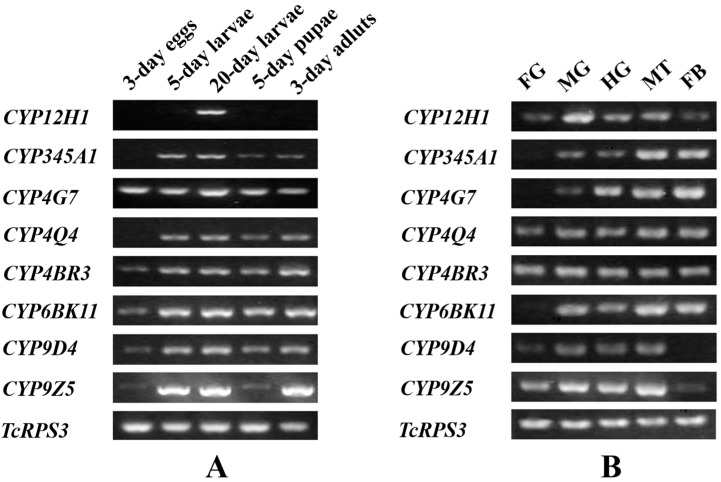
Stage-dependent (**A**) and tissue-dependent (**B**) expression patterns of eight selected CYP genes in *T. castaneum* (Georgia-1 strain). The expression profiles were evaluated by reverse transcription PCR (RT-PCR). The expression patterns of five different tissues, including foregut (FG), midgut (MG), hindgut (HG), Malpighian tubules (MT), fat bodies (FB) were derived from 20-day larvae, and *TcRPS3* was used as an internal reference gene.

For the stage-dependent expression pattern of the eight CYP genes, we found that almost all these genes were expressed in 20-day larvae, 5-day pupae and 3-day adults except *CYP12H1* which appeared to be only expressed in 20-day larvae (Figure 2A). The *CYP12H1* expression pattern is consistent with that of the insecticide resistant strain (QTC279) of *T. castaneum* reported by Zhu *et al.* [16]. On the other hand, *CYP9Z5* showed high expression in larval and adult stages but very low expression in egg and pupal stages. Its high expression appeared to associate with insect feeding. In contrast, *CYP12H1*, *CYP345A1* and *CYP4Q4* did not show detectable expression in eggs. However, the remaining five CYPs were constitutively expressed in all life stages. For the tissue-dependent expression pattern, *CYP12H1*, *CYP4Q4*, *CYP4BR3* and *CYP9Z5* were expressed in all the examined tissues (Figure 2B), and all the eight genes were expressed in midgut, hindgut and Malpighian tubules. However, the expression was undetectable for *CYP345A1*, *CYP4G7* and *CYP6BK11* in foregut and for *CYP9D4* in fat bodies.

Different expression patterns of CYP genes in different developmental stages of an insect suggest the diverse roles of these genes during the insect development [52]. For example, *CYP12H1* is only expressed in 20-day larvae, which implies its role restricted to this stage [16]. On the other hand, all the eight selected CYP genes were expressed in the midgut, Malpighian tubules and fat bodies (except *CYP9D4*), which are considered as major tissues involved in metabolism of xenobiotics in insects [53,54]. Therefore, such tissue-specific expression patterns of these CYP genes in *T. castaneum* may reflect their roles in metabolism of endogeneous and exogenous substances. Since all the eight CYP genes were expressed in 20-day larvae, we used this larval stage in our subsequent studies.

### 2.4. Selection of Insecticide Concentrations to Mediate Up-Regulation of CYP Genes

The lethal concentrations to kill 20% (LC_20_) and 50% (LC_50_) of the insect population, and their 95% confidence intervals (95% CI) for each of the four insecticides were evaluated in 20-day larvae (Table 2, Supplementary Figure S1). The order from the most to least toxic of the four insecticides is lambda-cyhalothrin, cypermethrin, imidacloprid and permethrin. Imidacloprid is a neonicotinoid whereas the remaining three are pyrethroids. Based on these results, we selected two concentrations approximate to the LC_20_ and one fourth of the LC_20_ of each insecticide to evaluate possible up-regulation of the eight CYP genes mediated by these insecticides. The approximate LC_20_ concentrations were 2 μg/mL for cypermethrin and lambda-cyhalothrin, and 16 μg/mL for permethrin and imidacloprid. The concentrations for the one fourth of approximate LC_20_ were 0.5 μg/mL for cypermethrin and lambda-cyhalothrin, and 4 μg/mL for permethrin and imidacloprid.

### 2.5. Evaluation of CYP Gene Up-Regulation by Insecticides

**Figure 3:**
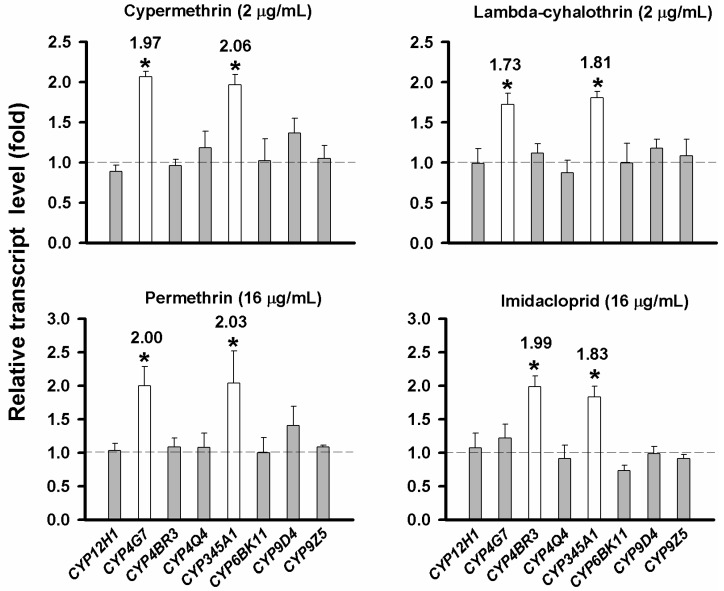
Up-regulation of CYP genes in *T. castaneum* after exposed to different insecticides. Dash lines represent relative transcript level of the control (larvae treated with the insecticide solvent only) as 1.0. The up-regulation fold was acquired by comparing the transcript levels of each CYP between the treated and the control insects. The CYP genes with a statistically significant up-regulation are marked with asterisks (Student’s *t* test, * *p* < 0.05).

In order to examine which CYP genes can be significantly up-regulated by insecticides, reverse transcription quantitative PCR (RT-qPCR) was performed to determine the change of transcript level for each of the eight CYP genes after the 20-day larvae were exposed to cypermethrin, lambda-cyhalothrin, permethrin or imidacloprid at their approximate LC_20_ concentrations for 24 h (Figure 3). *CYP345A1* was up-regulated by all the four insecticides tested, whereas *CYP4G7* was up-regulated by all the three pyrethroids but not by imidacloprid. However, *CYP4BR3* was up-regulated only by imidacloprid. Overall, only *CYP4G7* and *CYP345A1* can be up-regulated by the three pyrethroids, and only *CYP4BR3* and *CYP345A1* can be up-regulated by imidacloprid. The levels of the up-regulation range from 1.73–2.06-fold.

**Table 2:** Summary of the lethal concentrations to kill 20% (LC20) and 50% (LC_50_) of the insect population, and their 95% confidence intervals (95% CI) for each of the four insecticides determined in 20-day larvae of *T. castaneum* (Georgia-1 strain).

Insecticides	LC_20_, μg/mL (95% CI)	LC_50_, μg/mL (95% CI)	Slope ^a^	Intercept ^a^	χ^2^	*p* ^b^
Cypermethrin	2.27 (1.6–3.0)	7.77 (6.5–9.4)	3.66 ± 0.67	1.94 ± 0.94	5.13	1
Lambda-cyhalothrin	1.76 (1.6–3.3)	4.24 (3.6–5.0)	3.49 ± 0.40	2.98 ± 0.32	7.16	0.85
Permethrin	23.73 (15.3–32)	77.46 (63.6–94.9)	3.51 ± 0.80	1.63 ± 1.25	41.86	0.08
Imidacloprid	14.25 (12.0–16.5)	48.65 (44.9–53.4)	1.80 ± 0.49	2.02 ± 0.84	28.48	0.15

^a^ Slope and intercept were derived from the logarithm of concentration-probit mortality curve that generated by probit analysis; ^b^ *p*-Value > 0.05 indicates a significant fit between the observed and expected regression lines in a probit analysis.

### 2.6. Concentration- and Time-Dependent Effect on CYP Up-Regulation

**Figure 4:**
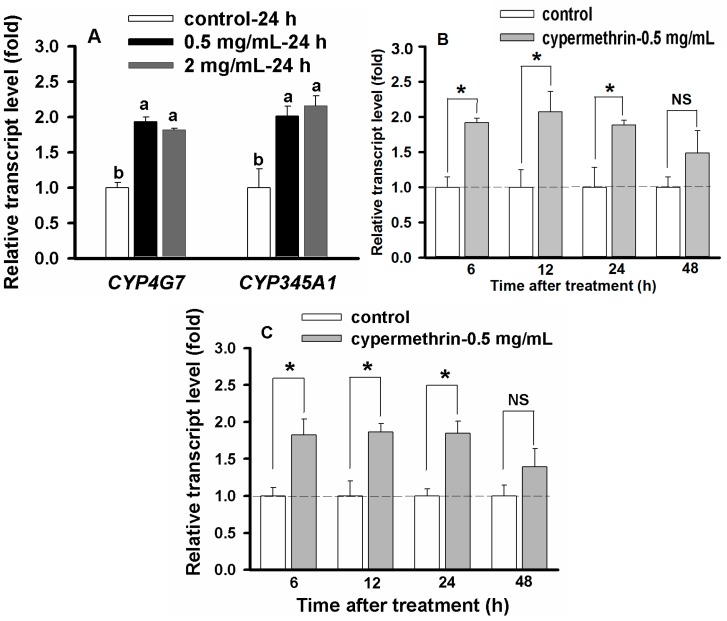
Cypermethrin concentration and time dependent up-regulation of *CYP4G7* and *CYP345A1* in 20-day larvae. Controls were normalized as 1.0, and the relative transcript levels of CYP genes were calculated based on their corresponding controls. (**A**) Cypermethrin concentration dependent up-regulation as measured at 2 and 0.5 μg/mL with the exposure time of 24 h. Different letters above the standard error bars indicate significant differences based on the one-way ANOVA followed by Tukey’s HSD multiple comparison test (*p* < 0.05); (**B**) Time dependent up-regulation of *CYP4G7* by cypermethrin (0.5 μg/mL) as measured at 6, 12, 24 and 48 h; and (**C**) Time dependent up-regulation of *CYP345A1* by cypermethrin (0.5 μg/mL) as measured at 6, 12, 24 and 48 h. Dash lines represent relative transcript level of the control (larvae treated with the insecticide solvent only) as 1.0. Statistical analysis was conducted to compare the expression levels between the control and the insecticide-treated insects within the same time duration by using Student’s *t* test. Asterisk above the standard error bars indicates significant difference whereas NS indicates no significant difference.

Because only *CYP4G7* and *CYP345A1* can be up-regulated by the three pyrethroids, and only *CYP4BR3* and *CYP345A1* can be up-regulated by imidacloprid (Figure 3), our studies on the concentration and time-dependent effect on the up-regulation focused on only three CYP genes (*CYP4G7*, *CYP345A1* and *CYP4BR3*). Two different concentrations of cypermethrin, 2 μg/mL (approximate LC_20_) and 0.5 μg/mL (one fourth of the approximate LC_20_), were used to expose 20-day larvae for 24 h followed by RT-qPCR analysis of *CYP4G7* and *CYP345A1*. The insects exposed to the two concentrations showed approximately 2-fold up-regulation in both genes, and the levels of the up-regulation did not show significant differences between the two insecticide concentrations (Figure 4A). Therefore, we used cypermethrin at 0.5 μg/mL for subsequent analyses.

The time-dependent effect on the CYP up-regulation was analyzed after 20-day larvae were exposed to cypermethrin at the concentration of 0.5 μg/mL for 6, 12, 24 and 48 h. Both *CYP4G7* (Figure 4B) and *CYP345A1* (Figure 4C) showed significant up-regulations compared to their corresponding controls (*i.e*., solvent exposures for the same durations) at 6, 12 and 24 h. However, the levels of such up-regulations began to decrease and did not show significant differences at 48 h compared with those of their controls for both *CYP4G7* and *CYP345A1*. These results indicated that the up-regulation of these two CYP genes by cypermethrin occurred at early stages of insecticide exposures (e.g., from 6–24 h). Based on this finding with cypermethrin, we used 6 h as the exposure time.

**Figure 5:**
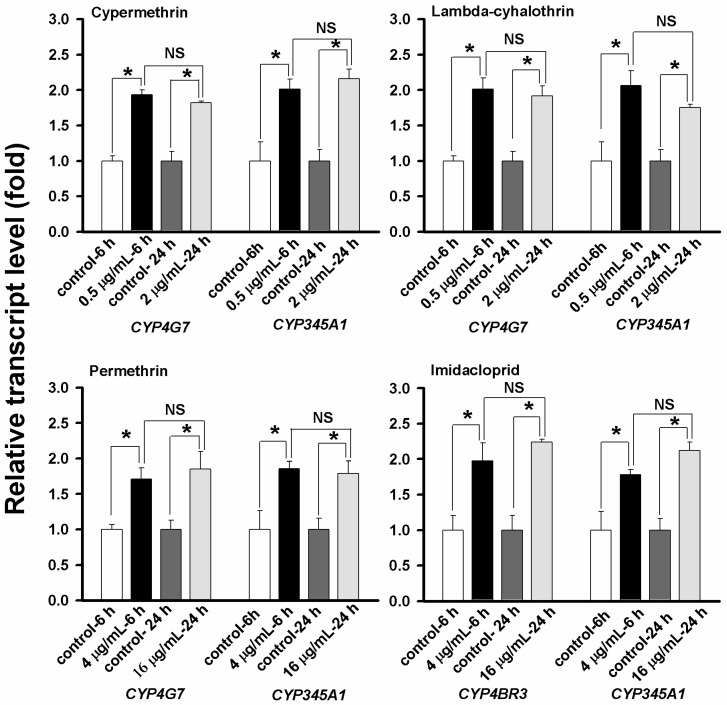
Insecticide concentration and time-dependent effect on the up-regulation of the CYP genes in 20-day larvae. The fold changes were also statistically compared between the two treatment combinations (*i.e*., 0.5 μg/mL for 6 h against 2 μg/mL for 24 h for cypermethrin and lambda-cyhalothrin, and 4 μg/mL for 6 h against 16 μg/mL for 24 h for permethrin and imidacloprid) by Student’s *t* test. An asterisk above the standard error bars indicates significant difference whereas NS indicates no significant difference.

Because a 6-h exposure to cypermethrin at 0.5 μg/mL resulted in up-regulations of both *CYP4G7* and *CYP345A1*, and such up-regulations were not significantly different from those with longer exposure times (*i.e*., 12, 24 and 48 h) and higher concentration of the insecticide (*i.e*., 2 μg/mL), we compared the up-regulation of the three genes between the exposure times (*i.e*., 6 and 24 h) and between two insecticide concentrations (*i.e*., 2 and 0.5 μg/mL for cypermethrin and lambda-cyhalothrin, and 16 and 4 μg/mL for permethrin and imidacloprid). As shown in Figure 5, all the exposures resulted in significant up-regulations of the CYP genes as compared with their corresponding controls. There were no statistical differences between the exposures at 0.5 μg/mL for 6 h and at 2 μg/mL for 24 h for cypermethrin and lambda-cyhalothrin, and between the exposures at 4 μg/mL for 6 h and at 16 μg/mL for 24 h for permethrin and imidacloprid.

### 2.7. Discussion

By using phylogenetic analysis and protein sequence comparisons of all the 143 CYP genes in *T. castaneum* retrieved from cytochrome P450 homepage (http://drnelson.uthsc.edu/CytochromeP450.html) along with those found in other insect species, we selected eight CYP genes, including *CYP4G7*, *CYP4Q4*, *CYP4BR3*, *CYP12H1*, *CYP6BK11*, *CYP9D4*, *CYP9Z5* and *CYP345A1*, from *T. castaneum* for detailed studies on their up-regulation mediated by different insecticides. These genes showed the highest identity levels at amino acid sequence levels with those known to be up-regulated by various chemicals including insecticides and/or involved in insecticide resistance in other insect species (Table 1). The overall amino acid identities of the CYPs between *T. castaneum* and other insect species ranged from 35%–54%, and seldom beyond 50%; only if the comparisons were made within the same subfamily. Although the strategies that we used to select representative CYP genes from *T. castaneum* for this study are justifiable, the overall low level of the amino acid sequence identities among the diverse CYPs from different insect species remains to be a challenge for selecting a manageable number of the CYP genes for detailed analyses. Nevertheless, our research provided useful information regarding the developmental stage and tissue-dependent expression patterns, the insecticide concentration and exposure time-dependent up-regulations, and the capability of different insecticides to mediate the up-regulation of various CYP genes representing eight different subfamilies in *T. castaneum.*

Our results showed that the up-regulation of *CYP4G7*, *CYP4BR3* and *CYP345A1* was relatively fast (6 h) and required only low concentrations of insecticides (e.g., one fourth of the LC_20_). The levels of the up-regulations in 20-day larvae of *T. castaneum* exposed to insecticides at one fourth of the approximate LC_20_ for 6 h were not significantly different from those of the larvae exposed to the same insecticides at approximate LC_20_ for 24 h. Our results suggested that relatively low concentrations of insecticides were effective for the up-regulation of CYP genes, and increasing the insecticide concentration may not necessarily enhance the up-regulation, possibly due to an increased toxic stress to the insects. However, several studies have shown that the up-regulation of a specific CYP gene can be influenced by chemical concentrations and exposure durations. In *D. melanogaster*, up-regulations of CYP genes increased gradually as the phenobarbital concentration and exposure duration increased [30]. In *P. xylostella*, Baek *et al.* [41] found that cypermethrin was able to up-regulate the CYPs transcription under different conditions. However, they found that the up-regulations were more effective when low sublethal concentrations and short exposure durations were used than those of high concentrations (e.g., LD_50_ or LC_50_) and long exposure duration. Reduced levels of up-regulations of CYP genes by insecticides at high concentrations could be due to increased stress of the insects caused by the insecticides. These results suggest that appropriate concentrations of an insecticide must be carefully pre-determined to evaluate the insecticide-mediated up-regulation of CYP genes in insects.

The level of the CYP up-regulation mediated by insecticides can also be affected by physiological status of insects. In *P. xylostella*, Bautista *et al.* [5] found that three out of six CYP genes were significantly up-regulated by permethrin in permethrin-susceptible strain, but only one of the six genes was moderately up-regulated by permethrin in permethrin-resistant strain although different concentrations of permethrin were used to expose the susceptible and resistant strains. In fact, four out of the six CYP genes in permethrin-resistant strain were significantly down-regulated by permethrin at 100 ppm. Furthermore, significant up-regulation of CYP genes by permethrin in the susceptible strain did not result in a decreased toxicity of permethrin to the insect. These results clearly demonstrated that the up-regulation of CYP genes by insecticides may not necessarily reflect their roles in insecticide detoxification. In fact, CYP genes involved in the detoxification of insecticides are often constitutively up-regulated in resistant insects. These genes may be less likely to be up-regulated by the insecticides.

Our results also indicated that there were no specific patterns related to the CYP gene families or subfamilies in their insecticide-mediated up-regulations in *T. castaneum*. First, only three out of the eight selected CYP genes (*i.e*., *CYP4G7*, *CYP4BR3* and *CYP345A1*) showed their up-regulation when the insects were exposed to each of the four insecticides, although these genes were selected from the CYP gene families (CYP12, CYP4, CYP6 and CYP9) known to be likely involved in insecticide metabolism and/or resistance [16]. None of the selected genes from the CYP6 and CYP9 families exhibited any insecticide-mediated up-regulation in this study. Secondly, previous studies showed that CYP genes which can be up-regulated or exhibited constitutive overexpression mediated by insecticides were mainly from CYP6B subfamily in *T. castaneum* [15,16]. However, our studies showed that the up-regulation of CYP genes was not restricted to a specific CYP family, as *CYP4G7* and *CYP4BR3* from CYP4 family, and *CYP345A1* from CYP345 family showed significant insecticide-mediated up-regulations.

The insecticide-mediated up-regulation of CYP genes in *T. castaneum* was insecticide specific. For example, both *CYP4G7* and *CYP4BR3* are from CYP4 family, but the former was significantly up-regulated by all three pyrethroids whereas the latter was uniquely up-regulated by imidacloprid. In contrast, *CYP345A1* was up-regulated by all the four insecticides, suggesting *CYP345A1* was responsive to a relatively broad spectrum of insecticides. However, the remaining five CYP genes were not significantly up-regulated by any of the four insecticides. Nevertheless, this does not necessarily mean that these CYP genes cannot be up-regulated by any insecticide. As a matter of fact, the up-regulation of CYP genes by specific insecticides has also been seen in other insect species. For instance, microarray analyses of the detoxification genes in *D. melanogaster* showed that spinosad, diazinon, nitenpyram, lufenuron and dicyclanil did not significantly increase the expression of any detoxification gene, but DDT induced only a single CYP gene (*CYP12D1*) among a total of 89 CYP genes [55]. In *Lymantria dispar*, 12 CYP genes exhibited different expression patterns (some up-regulated whereas others down-regulated) when the insects were exposed to different insecticides including deltamethrin, carbaryl and omethoate [56]. It was also noticed that the same CYP gene responded quite differently to different insecticides [56]. Thus, our results along with those from previous studies suggest that there is no general pattern for predicting the up-regulation of CYP genes based on the insecticide classifications.

## 3. Experimental Section

### 3.1. Insect Culture

The Georgia-1 (GA-1) insecticide-susceptible strain of *T. castaneum* was reared on whole-wheat flour containing 5% (*w*/*w*) of brewers’ yeast at 30 °C and 65% RH (relative humidity) in the growth chamber in Insect Toxicology Laboratory at Kansas State University (Manhattan, KS, USA).

### 3.2. Total RNA Isolation and First Strand cDNA Synthesis

Total RNA was isolated from each *T. castaneum* sample by using TRIzol reagent (Life Technologies, Carlsbad, CA, USA). Total RNA (2.0 μg) was first treated with DNase I (Fermentas, Glen Burnie, MD, USA) to remove potential genomic DNA contamination. The cDNAs were synthesized using EasyScript cDNA Synthesis SuperMix kit (Applied Biological Materials, Richmond, BC, Canada) with oligo(dT)18 as primer.

### 3.3. Phylogenetic Tree Construction

The deduced CYP amino acid sequences of *T. castaneum* and other insect species were retrieved from the National Center for Biotechnology Information (NCBI) (http://www.ncbi.nlm.nih.gov/) and Cytochrome P450 homepage (http://drnelson.uthsc.edu/CytochromeP450.html). The sequences were analyzed using ClustalW alignment with Molecular Evolutionary Genetic Analysis software version 5 (MEGA 5) (http://www.megasoftware net). The pair-wise alignments were performed with the gap opening penalty at 10 and the gap extension penalty at default 0.1. The multiple alignments were conducted with the gap opening penalty at 3 and the gap extension penalty at 1.8. The sites containing obvious missing data or alignment gaps were eliminated in a pair-wise manner. The phylogenetic tree was constructed using neighbor-joining algorithm with a total of 3000 bootstrap replications. Ultimately, the tree was created with cut off value of 50%. Sequence logos, which were predicted by WebLogo tool (http://weblogo.berkeley.edu/logo.cgi), depicted the conservation of amino acids in CYP heme-binding motif of each specific clade. The letter size is proportional to the degree of the conservation for amino acid residues of the motif.

### 3.4. Selection of Representative CYP Genes

The selections of CYP genes for studying insecticide-mediated up-regulation in *T. castaneum* were based on the representation of the genes in different CYP families, including CYP12, CYP4G, CYP4B, CYP4Q, CYP6B, CYP345, CYP9D and CYP9Z, and the identity levels of the deduced amino acid sequences of these genes compared with those of homologous genes known to be inducible by insecticides and other chemical substances and/or involved in insecticide resistance in other insect species. Only the genes showing lowest *E*-values and at least 35% identities from the same CYP family were selected from *T. castaneum* for further analyses.

### 3.5. Reverse Transcription Quantitative PCR (RT-qPCR) Analyses

The RT-qPCR was performed with EvaGreen qPCR MasterMix-iCycler (Applied Biological Materials) by using the Bio-Rad iCycler iQTM multi-color real-time PCR detection system (Bio-Rad Laboratories, Hercules, CA, USA). A gene encoding ribosomal protein S3, *TcRPS3*, was used as an internal reference [57]. Primers for RT-qPCR were designed by Beacon Designer™ (Table 3). RT-qPCR was performed with 3-step amplification protocol with 40 cycles of 95 °C for 15 s, 55 °C for 30 s and 70 °C for 30 s. At the end of the run, amplification specificity was verified by obtaining the dissociation curve, in which the samples were cooled to 55 °C after denaturing and then the melting curves were obtained by increasing 0.5 °C/10 s for each cycle with a total of 80 cycles until reaching 95 °C to denature the double-stranded DNA. The specificity of each reaction was evaluated based on the melting temperatures of the PCR products. The RT-qPCR was performed with three biological replications, and relative transcript levels of each gene were calculated according to the 2^−ΔΔ*C*t^ method [58,59].

**Table 3:** Primers used to analyze transcript levels of CYP genes in *T. castenuem*.

Primers	Sequence (5'–3')	Tm (°C)	Product Length (bp)
*TcCYP12H1-*F	AACCGCAAAAACTGATACGG	60.0	299
*TcCYP12H1-*R	ACCGGTCGTGTCTATTCCTG	60.0	
*TcCYP4G7*-F	CGCTGCCAACAGAGACATTA	60.0	207
*TcCYP4G7*-F	AATGACCCTGAAACCGTCAG	60.0	
*TcCYP4BR3*-F	CATCGGTTGTACCCTCCTGT	59.9	168
*TcCYP4BR3*-R	GAATCGGTCAGGGTCAAAGA	59.8	
*TcCYP4Q4-*F	TGGTTCCAATCACCCAATTT	60.0	203
*TcCYP4Q4-*R	TTTTTGCTCTTTGCGACCTT	59.5	
*TcCYP345A1-*F	TTTTTCGATTTTCGGTGGAG	60.0	120
*TcCYP345A1-*R	TTCGCGAAGGAAGTTGCTAT	60.0	
*TcCYP6BK11-*F	GTCAATTTGCGGAAACAGGT	60.1	167
*TcCYP6BK11-*R	CTACGTCCGTAAACCCGAAA	60.3	
*TcCYP9D4-*F	GTGGCACAACTAGCTCCACA	59.9	172
*TcCYP9D4-*R	GTTTTCCTTTACGGGCTTCC	60.0	
*TcCYP9Z5-*F	AGTCATGCAAAACTGCAACG	59.9	250
*TcCYP9Z5-*R	GTCCGGATTGGGGAAGTATT	60.0	
*TcRPS3-*F	CCGTCGTATTCGTGAATTGACTT	59.3	143
*TcRPS3-*R	TCTAAGAGACTCTGCTTGTGCAATG	60.8	

F: Forward; R: Reverse.

### 3.6. Stage and Tissue-Dependent Expression Patterns of CYP Genes

For analyses of developmental stage-dependent expression patterns, total RNA for each replication was isolated from 250–350 of 3-day old (3-day) eggs, 200–250 of 5-day larvae, 20–25 of 20-day larvae, 20–25 of 5-day pupae and 20–25 of 3-day adults. For analyses of tissue-dependent expression patterns, total RNA for each replication was isolated from each of five tissues (foregut, midgut, hindgut, Malpighian tubules and fat bodies) dissected from 80–100 of 20-day larvae. The selection of these tissues was mainly based on previous research showing abundant expressions of CYP genes in midgut, Malpighian tubules and fat bodies. Both the stage and tissue-dependent expression patterns of the eight CYP genes were examined using reverse transcription PCR (RT-PCR), which consisted of an initial denaturation at 95 °C for 3 min followed by 30 cycles of 95 °C for 30 s, 55 °C for 30 s and 70 °C for 30 s, and finished with a final extension step of 72 °C for 5 min. TcRPS3 was used as reference gene, and samples of 10-μL PCR products were analyzed on 2% agarose gel.

### 3.7. Insecticide Bioassay

Four insecticides, including cypermethrin (purity: 98%), lambda-cyhalothrin (96.8%), permethrin (97.5%) and imidacloprid (95.5%), were obtained from Chem Service (West Chester, PA, USA). Glass scintillation vials (20-mL) were internally coated with 0.5 mL of acetone containing each insecticide by using a RoTo-Torque rotator (Cole Parmer Instrument, Vernon Hills, IL, USA). At least five different concentrations of each insecticide, each with three replicates, were prepared for each bioassay. A group of 15 larvae (20-day) was transferred into each glass and larval mortality was assessed after the larvae were maintained in the vials at 30 °C and 65% RH (without flour) for 24 h. Larvae were considered dead if they were not able to move when gently touched with a brush. Data were analyzed by probit analysis using the procedure PROC PROBIT from SAS 9.3 (SAS Institute, Cary, NC, USA).

### 3.8. Evaluation of Up-Regulation of CYP Genes Mediated by Insecticides

The approximate LC_20_ of each of the four insecticides (2 μg/mL for either cypermethrin or lambda-cyhalothrin, 16 μg/mL for either permethrin or imidacloprid) was first used to expose 20-day larvae as described in Section 3.7. After the larvae were treated for 24 h, 4–5 surviving insects were collected form each replicate for total RNA extraction as described in Section 3.2. After we found each tested insecticide at LC_20_ could mediate significant up-regulations of the selected CYP genes, we included the one fourth of the approximate LC_20_ for each insecticide to compare the concentration-dependent effect of each insecticide on the CYP up-regulation. In addition, time-dependent effects were also evaluated using four different time points (6, 12, 24 and 48 h). After exposure, 4–5 surviving larvae were collected from each of 3 biological replicates for total RNA extraction, which was subsequently used to assess the expression levels of CYP genes using RT-qPCR as described in Section 3.5.

## 4. Conclusions

Many CYP genes are known for their rapid up-regulation in response to exposure to xenobiotics in insects. To date, however, limited information is available with respect to the relationship between the insecticide type, insecticide concentration, exposure duration and the up-regulated CYP genes. Our studies showed that *CYP4G7* and *CYP345A1* can be significantly up-regulated by cypermethrin, permethrin and lambda-cyhalothrin, whereas *CYP4BR3* and *CYP345A1* can be significantly up-regulated by imidacloprid in the eight selected CYP genes in 20-day larvae of *T. castenuem*. The levels of the up-regulation ranged from 1.73–2.06-folds. There were no significant differences in the level of up-regulation either between the two insecticide concentrations (*i.e*., approximate LC_20_ and the one fourth of the approximate LC_20_) or between the two insecticide exposure times (*i.e*., 24 and 6 h). Our study demonstrated that up-regulation of these CYP genes was rapid and only required low concentrations of insecticides. The up-regulation not only depended on the CYP genes but also the type of insecticides. Our results along with those from previous studies also indicated that there were no specific patterns for predicting the up-regulation of specific CYP gene families based on the insecticide classification.

## Supplementary Materials

Supplementary materials can be found at http://www.mdpi.com/1422-0067/16/01/2078/s1.
